# Doxorubicin-Induced Cardiotoxicity Through SIRT1 Loss Potentiates Overproduction of Exosomes in Cardiomyocytes

**DOI:** 10.3390/ijms252212376

**Published:** 2024-11-18

**Authors:** Shuai Zhang, Yu Yang, Xinchen Lv, Xue Zhou, Wangqian Zhao, Linfeng Meng, Hongfei Xu, Shaohua Zhu, Ying Wang

**Affiliations:** Department of Forensic Medicine, School of Basic Medicine, Soochow University, Suzhou 215123, China; 15225377016@163.com (S.Z.);

**Keywords:** doxorubicin-induced cardiotoxicity, cardiomyocytes, SIRT1, ATP6V1A, exosome secretion, mitochondrial-derived vesicles

## Abstract

Mutual interaction between doxorubicin (DOX) and cardiomyocytes is crucial for cardiotoxicity progression. Cardiomyocyte injury is an important pathological feature of DOX-induced cardiomyopathy, and its molecular pathogenesis is multifaceted. In addition to the direct toxic effects of DOX on cardiomyocytes, DOX-induced exosomes in the extracellular microenvironment also regulate the pathophysiological states of cardiomyocytes. However, the mechanisms by which DOX regulates exosome secretion and subsequent pathogenesis remain incompletely understood. Here, we found that DOX significantly increased exosome secretion from cardiomyocytes, and inhibiting this release could alleviate cardiomyocyte injury. DOX promoted exosome secretion by reducing cardiomyocyte silencing information regulator 1 (SIRT1) expression, exacerbating cardiotoxicity. DOX impaired lysosomal acidification in cardiomyocytes, reducing the degradation of intracellular multivesicular bodies (MVBs), resulting in an increase in MVB volume before fusing with the plasma membrane to release their contents. Mechanistically, SIRT1 loss inhibited lysosomal acidification by reducing the expression of the ATP6V1A subunit of the lysosomal vacuolar-type H+ ATPase (V-ATPase) proton pump. Overexpressing SIRT1 increased ATP6V1A expression, improved lysosomal acidification, inhibited exosome secretion, and thereby alleviated DOX-induced cardiotoxicity. Interestingly, DOX also induced mitochondrial-derived vesicle formation in cardiomyocytes, which may further increase the abundance of MVBs and promote exosome release. Collectively, this study identified SIRT1-mediated impairment of lysosomal acidification as a key mechanism underlying the increased exosome secretion from cardiomyocytes induced by DOX, providing new insights into DOX-induced cardiotoxicity pathogenesis.

## 1. Introduction

Doxorubicin (DOX) is a highly efficacious wide-spectrum anthracycline antibiotic widely used in the clinical treatment of various solid tumors and hematological malignancies [1,2]. However, its clinical utility is substantially limited by cardiotoxicity, leading to a range of cardiac problems, including left ventricular dysfunction, arrhythmia, congestive heart failure, and dilated cardiomyopathy [3,4]. Cardiomyocyte injury is a hallmark of DOX-induced cardiomyopathy. In cardiomyocytes, DOX generates reactive oxygen species (ROS) and damages lipids, DNA, and proteins. It also interferes with topoisomerase 2B (TOP2B), inducing double-strand DNA breaks. Furthermore, DOX triggers disruption of mitochondrial oxidative phosphorylation, permeability transition, and loss of membrane potential [5,6,7]. These effects ultimately promote cardiomyocyte death through pathways such as necroptosis, ferroptosis, pyroptosis, and apoptosis [7]. While previous studies have provided insights into DOX-induced cardiotoxicity, key gaps remain in fully elucidating the precise molecular pathogenesis.

SIRT1 is a nicotinamide adenosine dinucleotide (NAD+)-dependent deacetylase that maintains cardiac homeostasis [8]. SIRT1 alleviates cardiomyocyte injury by participating in pathways involving DOX-induced oxidative stress, DNA damage, mitochondrial dysfunction, and apoptosis [9,10]. Studies have shown that SIRT1 expression and activity are significantly reduced in DOX-induced cardiomyopathy, subsequently leading to myocardial fibrosis and cardiac dysfunction [11,12]. SIRT1 downregulation has also been linked to exosome secretion. Specifically, downregulating SIRT1 in adipose tissue stimulates exosome secretion in an autophagy-dependent manner, modulating obesity and insulin resistance via TLR4/NF-κB signaling [13]. Low SIRT1 expression in breast cancer cells [14,15] and podocytes in diabetic nephropathy [16] is associated with impaired lysosomal function and increased secretion of pathogenic exosome cargo, aggravating pathology. While SIRT1 appears important for both cardiomyocyte injury and exosome biogenesis, the relationship between SIRT1 inhibition, enhanced exosome secretion, and DOX-induced cardiotoxicity remains unclear.

Exosomes are nanoscale vesicles secreted by most cell types containing bioactive molecules such as nucleic acids, proteins, and lipids, which regulate the physiological and pathological conditions of recipient cells [17]. DOX treatment has been shown to increase exosome release [18], potentially mediating inter-cardiomyocyte communication [19]. Studies have shown that BALB/c mice administered with DOX produce higher levels of circulating exosomes in blood [18], and exosomes in bodily fluids can serve as biomarkers of DOX-induced cardiotoxicity [20]. Exosomes originate from intracellular membrane budding that forms multivesicular bodies (MVBs) [21]. MVBs contain cellular material exchanged from within the cell that either fuses with lysosomes within the cell or is transported along microtubules to fuse with the plasma membrane, releasing exosome content into the extracellular environment. Evidence indicates that DOX impairs lysosomal acidification and function in cardiomyocytes [22,23]. Lysosomal dysfunction could alter intercellular communication by preventing MVB degradation and increasing exosome release [14]. Elucidating the relationship between DOX effects on lysosomes and exosome biogenesis may provide novel insights into the molecular mechanisms of DOX-induced cardiotoxicity.

Given the established relationship between SIRT1, cardiomyocyte injury induced by DOX, and exosome secretion, we hypothesized a potential link between SIRT1 depletion and DOX-stimulated exosome release from cardiomyocytes. In this study, we found that DOX treatment induced excessive exosome production from cardiomyocytes, and these exosomes contributed to DOX-mediated cardiotoxicity. This occurred through SIRT1 loss, which inhibited lysosomal acidification and reduced fusion between MVBs and lysosomes. Consequently, an increased number of non-fused MVBs bound to the plasma membrane and were released as exosomes. In addition, DOX treatment increased mitochondrial-derived vesicle (MDV) production, which may have enriched the MVBs cargo composition and increased exosome release, exacerbating toxicity. Collectively, these results revealed a pathological mechanism by which DOX promoted exosome secretion through SIRT1 dysregulation, in turn exacerbating myocardial injury.

## 2. Results

### 2.1. Characterization of DOX-Induced Cardiotoxicity In Vivo and In Vitro

**Figure 1:**
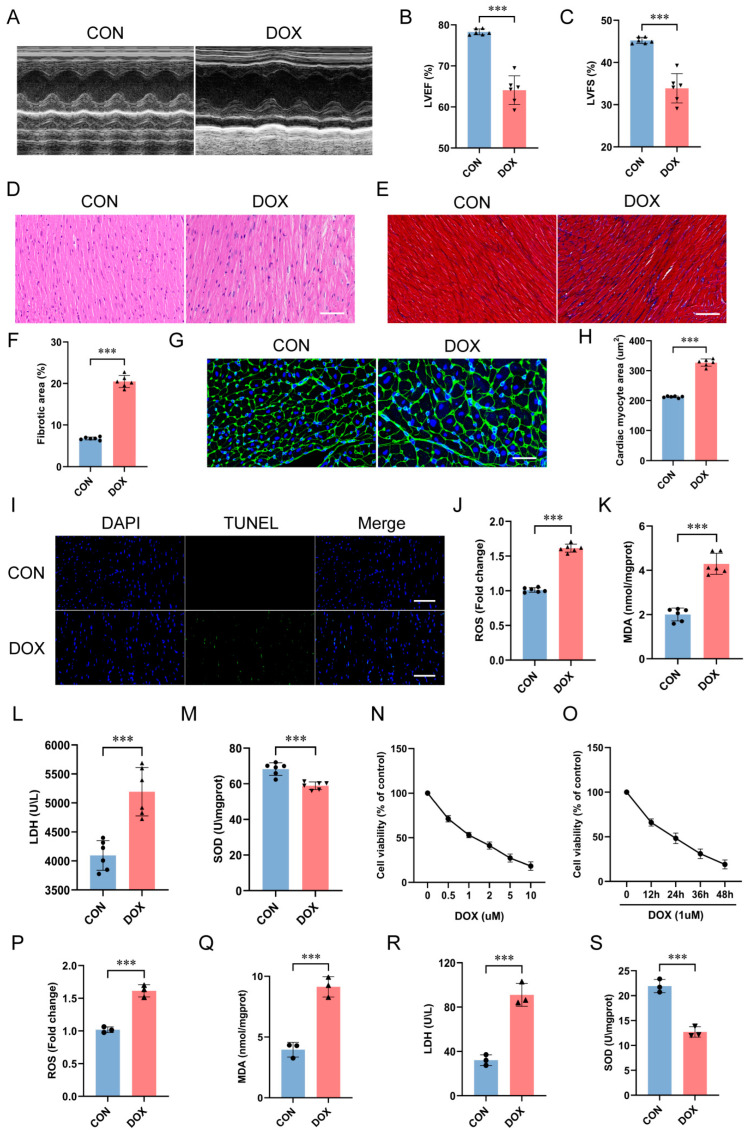
DOX−induced cardiotoxicity evaluated in vivo and in vitro. (**A**) Representative M−mode images of transthoracic echocardiography. (**B**,**C**) Quantification of (**B**) left ventricular ejection fraction (LVEF) and (**C**) left ventricular fractional shortening (LVFS) (*n* = 6 animals per group). (**D**) Representative H&E staining images of heart sections (scale bar: 50 μm). (**E**) Representative Masson’s trichrome staining images of heart sections (*n* = 6 animals per group) (scale bar: 50 μm). (**F**) Quantification of fibrotic area. (**G**) Representative images of wheat germ agglutinin (WGA) staining in heart sections (*n* = 6 animals per group) (scale bar: 50 μm). Nuclei stained with 4′,6−diamidino−2−phenylindole (DAPI, blue) and WGA represented cardiomyocyte borders (green). (**H**) Quantification of cardiomyocyte hypertrophy. (**I**) Representative images of TdT−mediated dUTP nick−end labeling (TUNEL) staining (green) of heart sections (*n* = 6 animals per group) (scale bar: 50 μm). Apoptotic cell nuclei appear in green fluorescence, and normal nuclei appear in blue fluorescence (DAPI). (**J**–**M**) Cardiac reactive oxygen species (ROS), malondialdehyde (MDA), serum lactate dehydrogenase (LDH), and cardiac superoxide dismutase (SOD) levels (*n* = 6 animals per group). (N, O) H9c2 cell viability following (**N**) gradient DOX concentrations and (**O**) treatment durations (*n* = 3 independent cell culture experiments). (**P**–**S**) ROS, MDA, supernatant LDH, and SOD levels in H9c2 cells (*n* = 3 independent cell culture experiments). Data are presented as mean ± SD. *** *p* < 0.001 vs. control group.

To characterize DOX-induced cardiotoxicity, we used a well-established mouse model of chronic DOX treatment at 5 mg/kg, administered once per week for four treatments [24]. Echocardiography revealed that DOX significantly reduced the left ventricular ejection fraction (LVEF) and fractional shortening (LVFS), demonstrating impaired cardiac function (Figure 1A–C). Histological analyses revealed severe disruptions to myocardial architecture in DOX-treated hearts, characterized by disorganized myocardial fibers (Figure 1D), interstitial fibrosis (Figure 1E,F), cardiomyocyte hypertrophy (Figure 1G,H), and increased necrosis (Figure 1I). Following DOX treatment, cardiac oxidative stress markers reactive oxygen species (ROS; Figure 1J), malondialdehyde (MDA; Figure 1K), and serum cardiac injury marker lactate dehydrogenase (LDH; Figure 1L) were significantly increased, while the level of cardiac superoxide dismutase (SOD), an enzyme catalyzing peroxides reduction, was significantly decreased (Figure 1M). Collectively, these results demonstrated that DOX impaired both cardiac function and structure, inducing myocardial damage indicative of cardiotoxicity.

To elucidate the underlying cellular and molecular mechanisms, we used the H9c2 cardiomyoblast cell line. H9c2 cells are derived from rat cardiac tissue and are widely used in cardiovascular research due to their retention of key cardiomyocyte properties, including skeletal muscle and myocardial characteristics. Cells were treated with DOX at gradient concentrations of 0, 0.5, 1, 2, 5, and 10 μM for 24 h (Figure 1N). DOX treatment significantly decreased cell viability in a dose-dependent manner, with cell viability halved at a concentration of 1 μM. Cells were then cultured with 1 μM of DOX for durations of 0, 12, 24, 36, and 48 h (Figure 1O). DOX significantly reduced cell viability in a time-dependent manner. Based on results from the cell viability, 1 μM of DOX for 24 h was selected for subsequent in vitro experiments to investigate toxicity mechanisms. Following DOX treatment, oxidative stress markers ROS (Figure 1P) and MDA (Figure 1Q), as well as the cell damage marker LDH (Figure 1R), were significantly increased in H9c2 cells, while the activity of peroxidase SOD was significantly decreased (Figure 1S). Thus, DOX exposure induced cardiotoxicity through cardiomyocyte injury in a modulated manner.

### 2.2. GW4869 Alleviated Oxidative Stress and Cardiac Dysfunction by Inhibiting DOX-Stimulated Exosome Release

**Figure 2:**
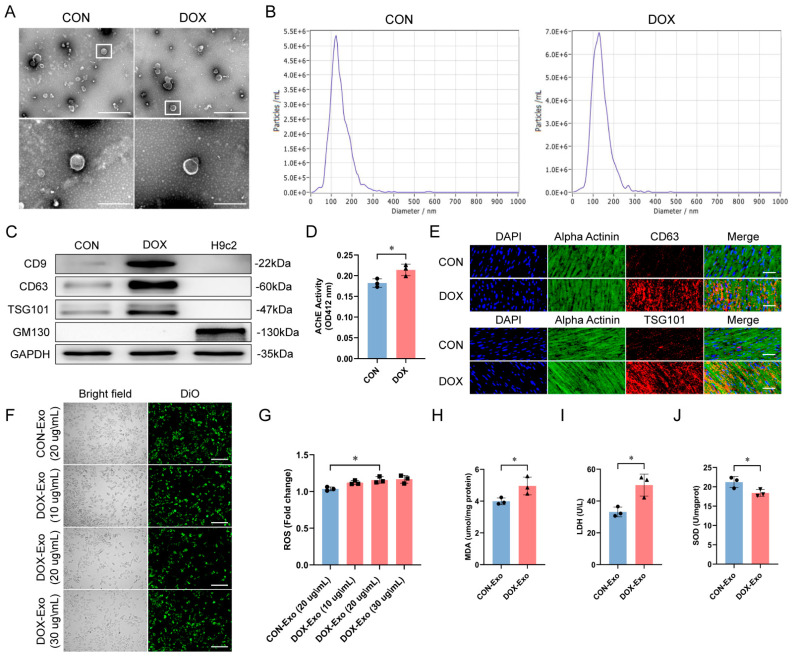
DOX−induced exosome secretion from cardiomyocytes. (**A**) Transmission electron micrographs of exosomes derived from H9c2 cells in control and DOX−treated groups (scale bar: 1 μm and 200 nm). (**B**) Nanoparticle tracking analysis of H9c2 cell−derived exosome size distribution. (**C**) Western blot analysis of exosome−specific markers CD9, CD63, and TSG101 and the non-exosomal marker GM130 in H9c2 cell−derived exosomes. Whole−cell lysates were used as controls. (**D**) Exosome concentration measured using acetylcholine esterase (AChE) activity (*n* = 3 independent cell culture experiments). (**E**) Immunofluorescence staining for CD63 and TSG101 (red) in heart sections from control and DOX−treated groups (scale bar: 50 μm). Sarcomeric alpha−actinin labeled cardiomyocytes (green). Nuclei stained with DAPI (blue). (**F**) Uptake of 3,3′−dioctadecyloxacarbocyanine perchlorate (DiO, green)−labeled exosomes by H9c2 cells (scale bar: 50 μm). (**G**–**J**) ROS, MDA, supernatant LDH, and SOD levels in H9c2 cells after exposure to exosomes from control and DOX−treated groups (*n* = 3 independent cell culture experiments). Data are presented as mean ± SD. * *p* < 0.05 vs. control group.

In investigating the effects of DOX in vitro, we found that DOX treatment stimulated exosome secretion from H9c2 cells. Exosome release was visualized using transmission electron microscopy (TEM) and nanoparticle tracking analysis (NTA). TEM revealed a typical cup-like structure vesicle with a size of approximately 100 nm released from H9c2 cells (Figure 2A). NTA measured these vesicles at around 120 nm (Figure 2B), consistent with the size of exosomes. Western blot analysis showed that the expression of the exosomal markers CD9, CD63, and TSG101 was increased in DOX-treated H9c2 cells (Figure 2C). The quantity of exosomes in cell culture supernatants was indirectly estimated by measuring acetylcholine esterase (AChE) activity, as AChE is enriched in exosomal membranes [25,26]. AChE analysis revealed that the level of AChE activity was increased (Figure 2D). In addition, immunofluorescence staining showed increased expression of the cardiomyocyte exosome-specific markers CD63 and TSG101 in cardiac tissue from DOX-treated mice (Figure 2E), indicating an increase in the production of cardiomyocyte-derived exosomes. Therefore, DOX induced H9c2 cells and mouse cardiomyocytes to secrete exosomes.

To evaluate whether these exosomes have proinflammatory effects, we isolated exosomes from supernatants of the control and DOX-treated H9c2 cells. Isolated exosomes were fluorescently labeled with 3,3′-dioctadecyloxacarbocyanine perchlorate (DiO) and added to untreated H9c2 cells at concentrations of 10, 20, and 30 ug/mL, respectively, to assess cellular exosome uptake (Figure 2F). Exosomes at 20 ug/mL from DOX-treated cells significantly increased ROS production in H9c2 cells compared to cells receiving exosomes from control cells, indicating enhanced oxidative stress upon cellular uptake of DOX-derived exosomes (Figure 2G). Consistent with this, cells exposed to DOX-derived exosomes exhibited elevated levels of MDA and LDH (Figure 2H,I), as well as decreased SOD activity (Figure 2J), compared to controls. These effects suggested that exosomes secreted from DOX-treated cells may contribute to oxidative stress mechanisms when taken up by recipient cardiomyocytes.

**Figure 3:**
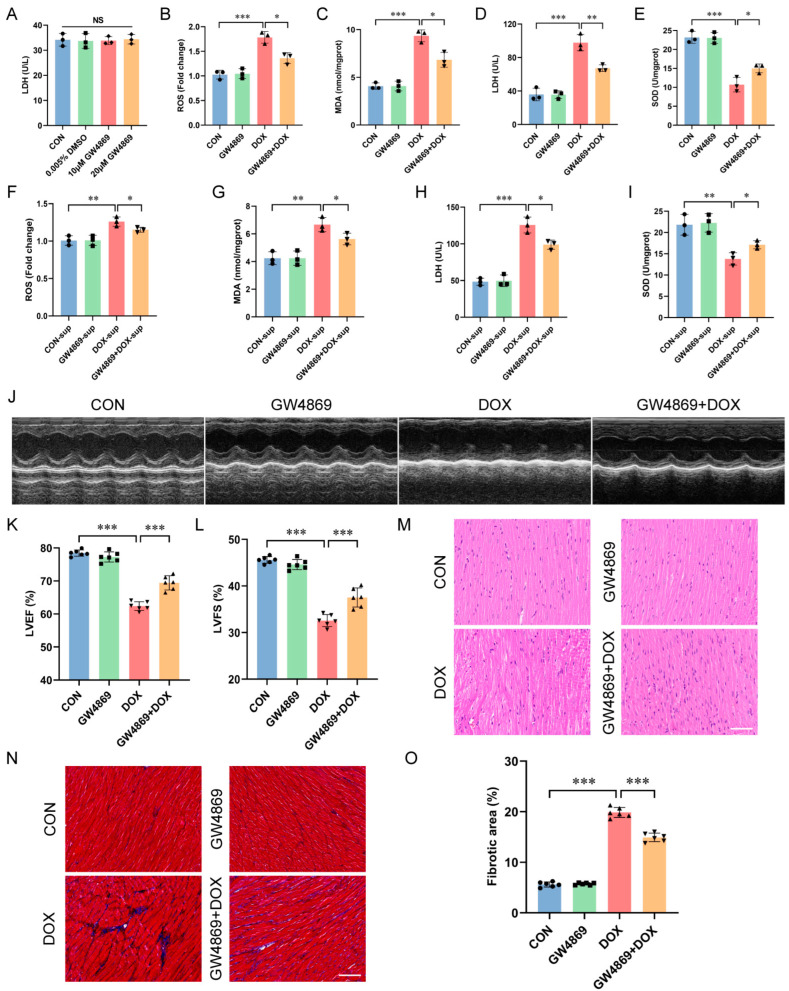

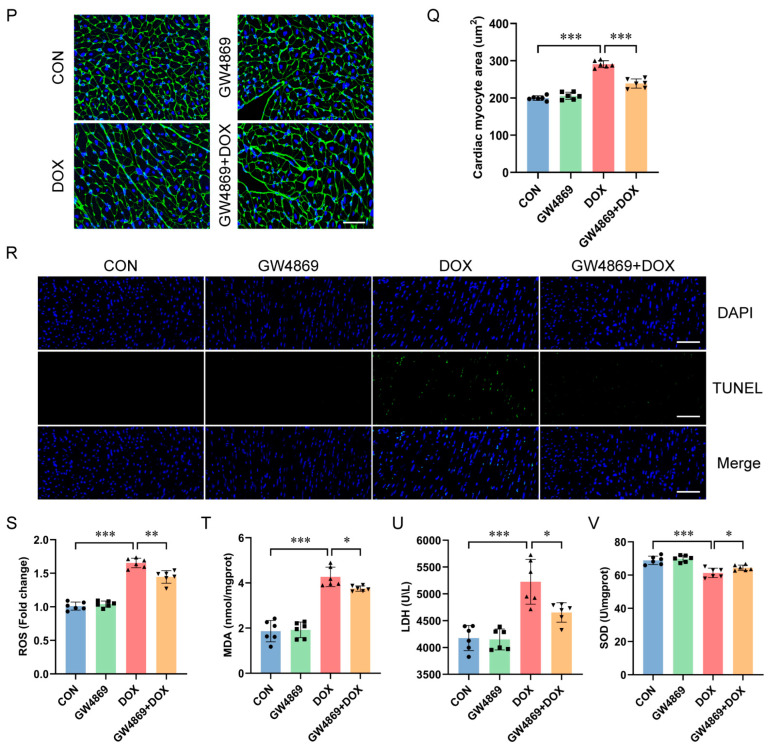
GW4869 attenuated DOX−induced cardiotoxicity. (**A**) LDH levels in H9c2 cells showed 0.005% DMSO and GW4869 at 10 μM and 20 μM were not cytotoxic compared to control (*n* = 3 independent cell culture experiments). (**B**–**E**) ROS, MDA, supernatant LDH, and SOD levels in H9c2 cells (*n* = 3 independent cell culture experiments). (**F**–**I**) ROS, MDA, supernatant LDH, and SOD levels in H9c2 cells after treatment with culture supernatants from the CON, GW4869, DOX, and GW4869 + DOX groups (*n* = 3 independent cell culture experiments). (**J**) Representative M−mode images of transthoracic echocardiography. (**K**,**L**) Quantification of (**K**) LVEF and (**L**) LVFS (*n* = 6 animals per group). (**M**) H&E staining of heart sections (scale bar: 50 μm). (**N**) Representative Masson’s trichrome staining images of heart sections (*n* = 6 animals per group) (scale bar: 50 μm). (**O**) Quantification of fibrotic area. (**P**) Representative WGA staining images of heart sections (*n* = 6 animals per group) (scale bar: 50 μm). Nuclei stained with DAPI (blue) and WGA represented cardiomyocyte borders (green). (**Q**) Quantification of cardiomyocyte hypertrophy. (**R**) Representative TUNEL staining images of heart sections (*n* = 6 animals per group) (scale bar: 50 μm). Apoptotic cell nuclei appear in green fluorescence, and normal nuclei appear in blue fluorescence (DAPI). (**S**–**V**) Cardiac ROS, MDA, serum LDH, and cardiac SOD levels (*n* = 6 animals per group). Data are presented as mean ± SD. NS, not significant (*p* > 0.05), * *p* < 0.05, ** *p* < 0.01 and *** *p* < 0.001 vs. control group.

To investigate whether inhibiting exosome release protected against DOX-induced cardiotoxicity, we used the exosome biogenesis and release inhibitor GW4869 at 10 μM and 20 μM [26]. An LDH assay showed that 0.005% DMSO and GW4869 were not cytotoxic to H9c2 cells (Figure 3A). Given the potent inhibitory effects of GW4869 at high concentrations, 20 μM was selected for subsequent in vitro experiments. In H9c2 cells, DOX exposure increased oxidative damage, as evidenced by elevated ROS (Figure 3B), MDA (Figure 3C), and LDH (Figure 3D) levels with decreased SOD activity (Figure 3E). However, GW4869 mitigated DOX-induced changes in these markers. Meanwhile, equal volumes of culture supernatants from the CON, GW4869, DOX, and GW4869 + DOX groups, each containing exosomes, were added to normal H9c2 cells (Figure 3F–I). Compared to the DOX group, H9c2 cells incubated with the supernatant from the GW4869 + DOX group showed significantly reduced oxidative damage. We then evaluated the effect of GW4869 on inhibiting DOX-mediated exosome release and cardiac damage in vivo. GW4869 co-administration significantly attenuated DOX-induced cardiac dysfunction (Figure 3J–L), histopathological alterations (Figure 3M), myocardial fibrosis (Figure 3N,O), cardiomyocyte hypertrophy (Figure 3P,Q), and necrosis (Figure 3R) in mice. GW4869 also reversed DOX-elicited elevations in cardiac oxidative markers ROS (Figure 3S), MDA (Figure 3T), and serum cardiac injury marker LDH (Figure 3U), and decrease in cardiac peroxidase SOD activity (Figure 3V). Collectively, these data indicated that inhibition of exosome secretion by GW4869 protected against DOX-induced cardiotoxicity by reducing myocardial damage and preserving cardiac function in vitro and in vivo.

### 2.3. SIRT1 Overexpression Protected Against DOX-Induced Cardiac Damage and Dysfunction by Inhibiting Exosome Release

**Figure 4:**
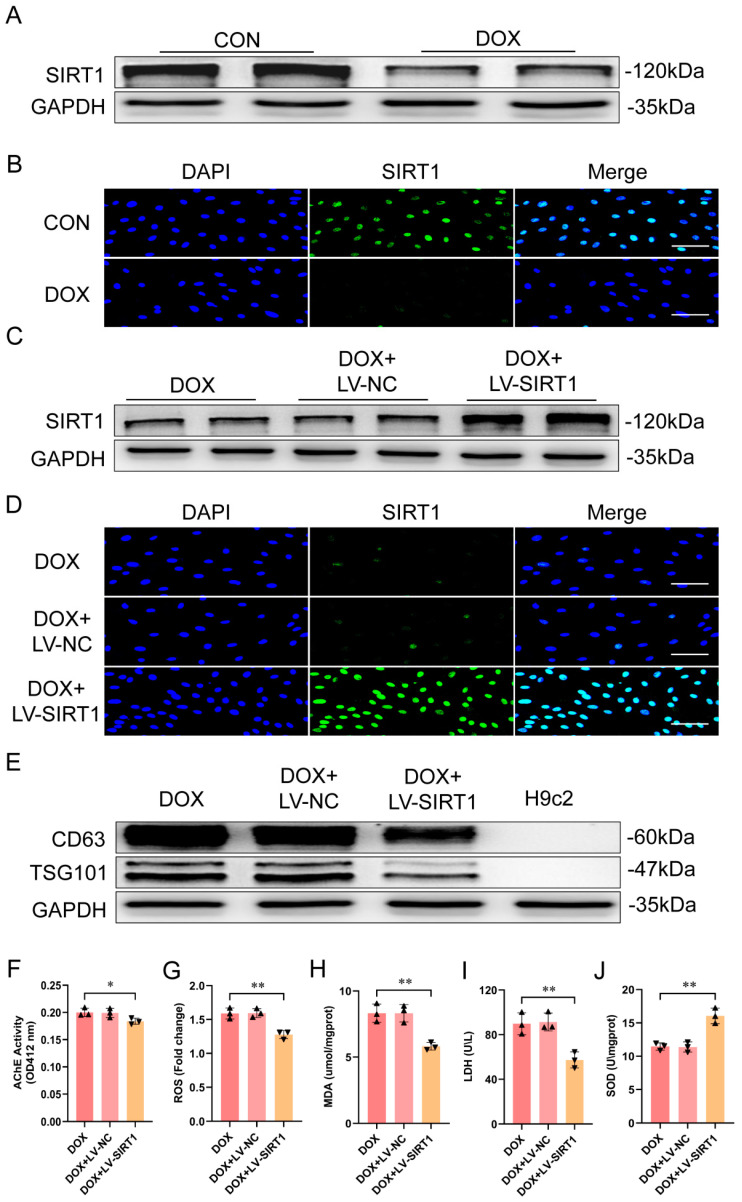
DOX affected exosome secretion through SIRT1 in cardiomyocytes. (**A**) SIRT1 protein expression levels in H9c2 cells as measured using western blot. (**B**) Immunofluorescence staining of SIRT1 (green) in H9c2 cells (*n* = 3 independent cell culture experiments) (scale bar: 50 μm). (**C**) SIRT1 overexpression in H9c2 cells as measured using western blot. (**D**) Immunofluorescence staining of SIRT1 (green) in H9c2 cells (*n* = 3 independent cell culture experiments) (scale bar: 50 μm). (**E**) Western blot analysis of exosomal markers CD63 and TSG101 in H9c2 cell−derived exosomes. (**F**) Exosome concentration measured using AChE activity (*n* = 3 independent cell culture experiments). (**G**–**J**) ROS, MDA, supernatant LDH, and SOD levels in H9c2 cells (*n* = 3 independent cell culture experiments). Data are presented as mean ± SD. * *p* < 0.05 and ** *p* < 0.01 vs. control group.

As exosome release is implicated in the pathogenesis of DOX-induced cardiotoxicity, we further elucidated the mechanisms underlying DOX-induced exosome secretion. Given the key role of SIRT1 in maintaining cardiac homeostasis and its close association with exosome release [15,27], we evaluated the correlation between SIRT1 expression and DOX induction of exosome secretion in H9c2 cells. Western blot and immunofluorescence staining showed that SIRT1 expression was decreased in H9c2 cells after DOX treatment (Figure 4A,B). H9c2 cells were treated with lentivirus overexpressing SIRT1 (LV-SIRT1) or a negative control (LV-NC) following DOX treatment (Figure 4C,D). Western blot revealed reduced exosomal markers CD63 and TSG101 in exosomes from DOX-treated, SIRT1-overexpressing H9c2 cells (Figure 4E). AChE activity was also decreased (Figure 4F). In DOX-treated, SIRT1-overexpressing H9c2 cells, SIRT1 decreased ROS, MDA, and supernatant LDH levels while increasing SOD activity (Figure 4G–J).

**Figure 5:**
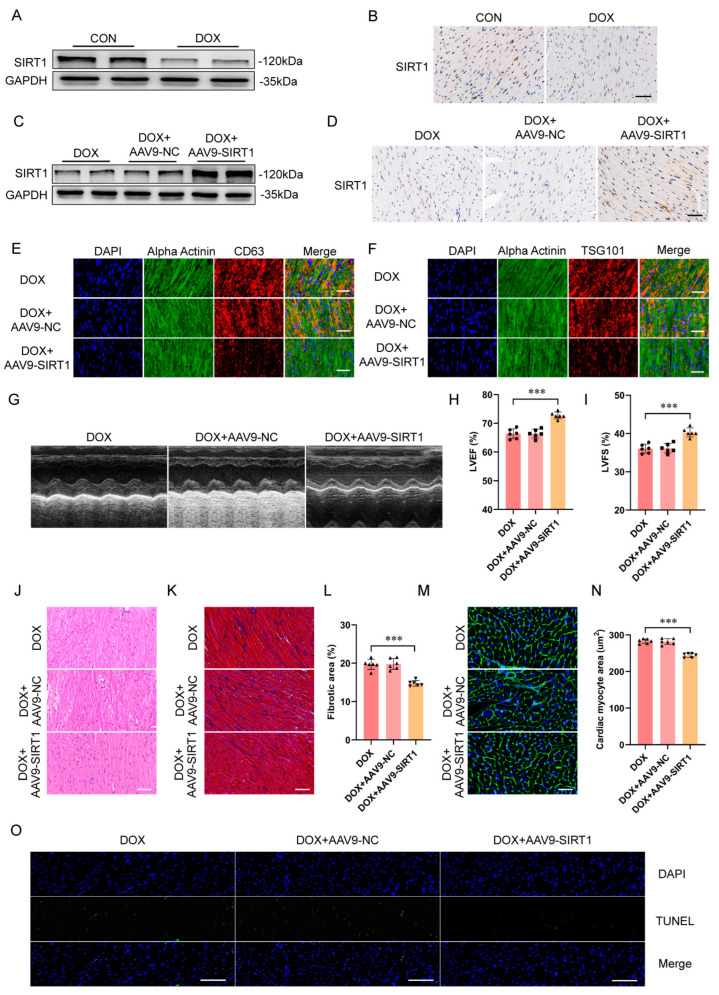

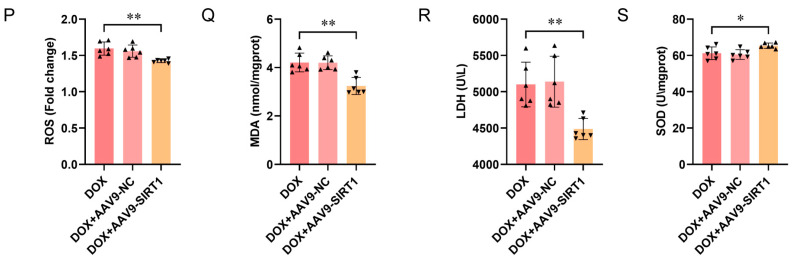
Overexpression of SIRT1 attenuated DOX−induced cardiotoxicity. (**A**) SIRT1 protein expression levels in mouse hearts as measured using western blot. (**B**) Immunohistochemistry staining of SIRT1 (brown) in heart sections (scale bar: 50 μm). (**C**) SIRT1 overexpression in mouse hearts as measured using western blot. (**D**) Immunohistochemistry staining of SIRT1 overexpression (brown) in heart sections (scale bar: 50 μm). (**E**,**F**) Immunofluorescence staining of exosome markers (**E**) CD63 and (**F**) TSG101 (red) in heart sections after SIRT1 overexpression (scale bar: 25 μm). Sarcomeric alpha−actinin labeled cardiomyocytes (green). Nuclei stained with DAPI (blue). (**G**) Representative M−mode images of transthoracic echocardiography for each group 2 weeks after AAV9−SIRT1 treatment. (**H**,**I**) Quantification of (**H**) LVEF and (**I**) LVFS (*n* = 6 animals per group). (**J**) Representative images of H&E staining in heart sections (scale bar: 50 μm). (**K**) Representative images of Masson’s trichrome staining in heart sections (*n* = 6 animals per group) (scale bar: 50 μm). (**L**) Quantification of fibrotic area. (**M**) Representative images of WGA staining in heart sections (*n* = 6 animals per group) (scale bar: 50 μm). Nuclei stained with DAPI (blue) and WGA represented cardiomyocyte borders (green). (**N**) Quantification of cardiomyocyte hypertrophy. (**O**) Representative images of TUNEL staining of heart sections (*n* = 6 animals per group) (scale bar: 50 μm). Apoptotic cell nuclei appear in green fluorescence, and normal nuclei appear in blue fluorescence (DAPI). (**P**–**S**) Cardiac ROS, MDA, serum LDH, and cardiac SOD levels. Data are presented as mean ± SD. * *p* < 0.05, ** *p* < 0.01 and *** *p* < 0.001 vs. control group.

Similarly, western blot and immunohistochemistry showed that SIRT1 expression was decreased in mouse hearts after DOX treatment (Figure 5A,B). To overexpress SIRT1 in mouse myocardium, recombinant adeno-associated virus serum type nine carrying SIRT1 (AAV9-SIRT1) or a negative control (AAV9-NC) was injected via the tail vein (Figure 5C,D). Immunofluorescence analysis revealed that SIRT1 overexpression reduced the DOX-induced upregulation of exosomal markers CD63 and TSG101 in mouse cardiomyocytes (Figure 5E,F). SIRT1 overexpression also significantly attenuated DOX-induced impairments in cardiac function as measured using echocardiography (Figure 5G–I) as well as histomorphological changes (Figure 5J), myocardial fibrosis (Figure 5K,L), cardiomyocyte hypertrophy (Figure 5M,N), and necrosis (Figure 5O). Furthermore, SIRT1 overexpression significantly reversed the DOX-elicited increase in myocardial ROS (Figure 5P), MDA (Figure 5Q), and serum LDH levels (Figure 5R) while preserving myocardial SOD activity (Figure 5S). Taken together, both in vivo and in vitro findings suggested that DOX-induced cardiotoxicity by exosome release was through downregulating SIRT1 expression, thereby potentially exacerbating cardiotoxicity.

### 2.4. Downregulation of SIRT1-ATP6V1A Mediated DOX-Induced Loss of Lysosomal Acidification

**Figure 6:**
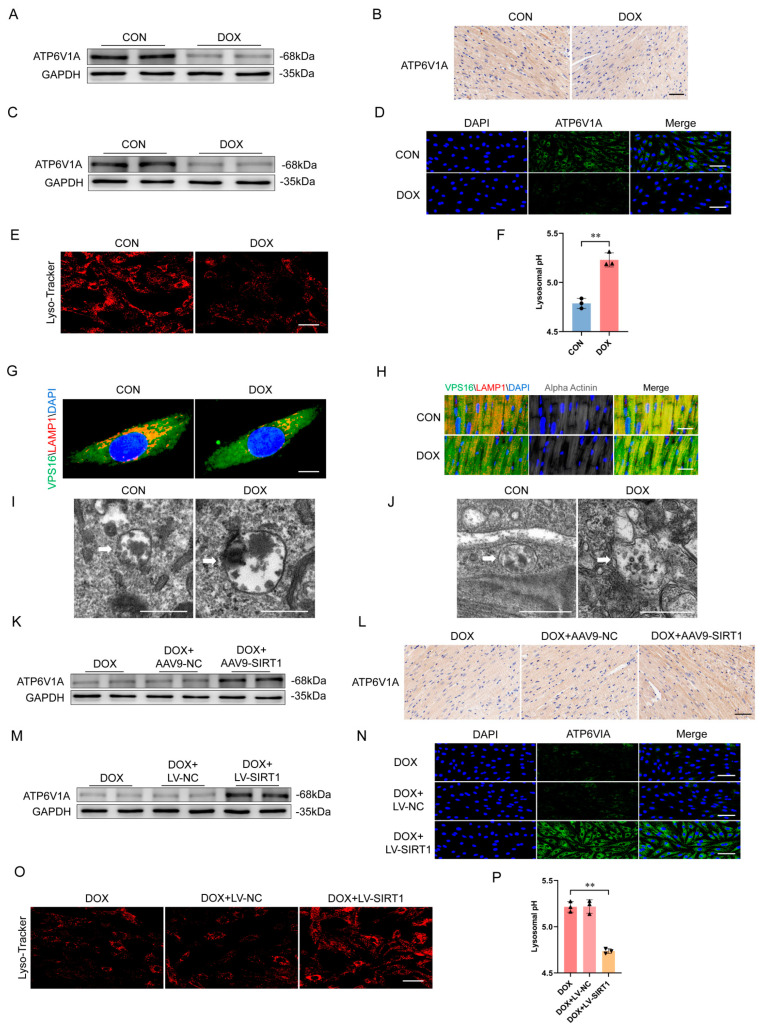

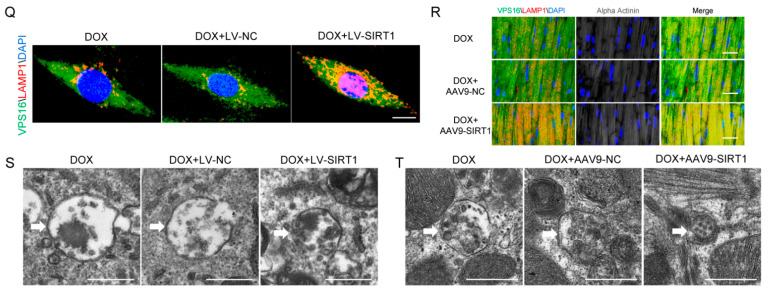
DOX inhibited lysosomal acidification by regulating ATP6V1A expression through SIRT1 in cardiomyocytes. (**A**) Western blot analysis of ATP6V1A protein levels in mouse hearts. (**B**) Representative immunohistochemistry staining of ATP6V1A (brown) staining in heart sections (scale bar: 50 μm). (**C**) Western blot of ATP6V1A expression in H9c2 cells. (**D**) Representative immunofluorescence staining of ATP6V1A (green) in H9c2 cells (scale bar: 50 μm). (**E**) DOX treatment for 24 h reduced Lysotracker Red−positive puncta (Cat#C1046, Beyotime Biotechnology, Haimen, China) in H9c2 cell cytoplasm (scale bar: 50 μm). (**F**) DOX increased lysosomal pH as measured using Dextran, Oregon Green 514 (*n* = 3 independent cell culture experiments). (**G**,**H**) Representative double immunofluorescence images of VPS16 (green) and LAMP−1 (red) co−localization in (**G**) H9c2 cells and (**H**) mouse hearts, indicating lysosome−MVBs interaction (scale bar: 10 μm and 25 μm). Sarcomeric alpha−actinin labeled cardiomyocytes (gray, central panels). Nuclei stained with DAPI (blue). (**I**,**J**) Transmission electron micrographs showing MVBs in (**I**) H9c2 cells and (**J**) mouse hearts (scale bar: 500 nm). The white arrows pointed to the MVBs. (**K**–**N**) SIRT1 overexpression restored ATP6V1A expression in mouse hearts and H9c2 cells as measured using western blot and immunofluorescence staining. (**O**,**P**) SIRT1 increased (**O**) Lysotracker Red−positive puncta (scale bar: 50 μm) and decreased (**P**) lysosomal pH (*n* = 3 independent cell culture experiments). (**Q**,**R**) SIRT1 enhanced VPS16 (green) and LAMP−1 (red) co−localization in (**Q**) H9c2 cells (scale bar: 10 μm) and (R) mouse hearts (scale bar: 25 μm). Sarcomeric alpha−actinin labeled cardiomyocytes (gray, central panels). Nuclei stained with DAPI (blue). (**S**,**T**) Transmission electron micrographs showing MVBs in (**S**) H9c2 cells and (**T**) mouse hearts (scale bar: 500 nm). The white arrows pointed to the MVBs. Data are presented as mean ± SD. ** *p* < 0.01 vs. control group.

Given that SIRT1 regulates lysosomal acidification and MVB degradation by modulating the expression of the ATP6V1A subunit of the vacuolar-type H+ ATPase (V-ATPase) proton pump to promote exosome biogenesis [14], we elucidated the mechanism by which DOX affected exosome secretion from cardiomyocytes through SIRT1. We found that DOX treatment reduced ATP6V1A expression in mouse hearts (Figure 6A,B) and H9c2 cells (Figure 6C,D). DOX also impaired lysosomal acidification in H9c2 cells (Figure 6E,F). In addition, co-localization of the MVB marker VPS16 (green) and lysosome marker LAMP1 (red), represented by yellow puncta, was reduced by DOX treatment in cardiomyocytes in vivo (Figure 6G) and in vitro (Figure 6H). Furthermore, the volume of MVBs was increased in H9c2 cells and mouse hearts in the DOX-treated group compared with the control group (Figure 6I,J). In contrast, SIRT1 overexpression restored ATP6V1A expression in vivo (Figure 6K,L) and in vitro (Figure 6M,N), rescued lysosomal acidification function in H9c2 cells (Figure 6O,P), and increased co-localization of VPS16 and LAMP1 (larger yellow spots) in vitro (Figure 6Q) and in vivo (Figure 6R). In addition, SIRT1 overexpression decreased the volume of MVBs in both H9c2 cells and mouse hearts (Figure 6S, T).

**Figure 7:**
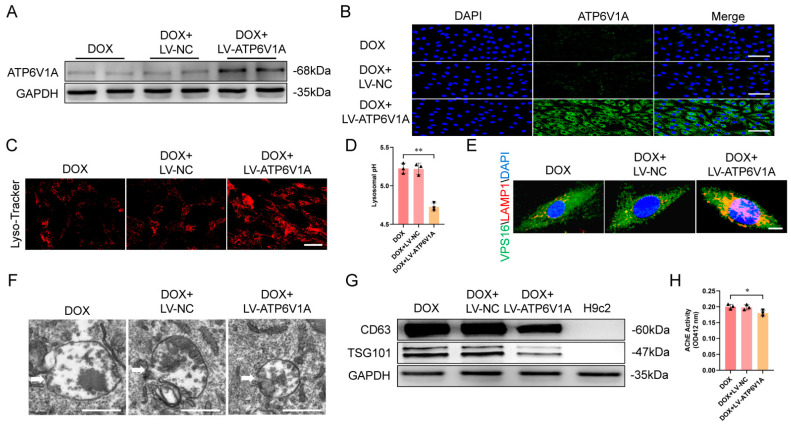
Overexpression of ATP6V1A inhibited exosome secretion. (**A**) Western blot analysis of ATP6V1A protein levels in H9c2 cells. (**B**) Representative immunofluorescence staining of ATP6V1A (green) in H9c2 cells (scale bar: 50 μm). (**C**) ATP6V1A overexpression increased Lysotracker Red−positive puncta in H9c2 cell cytoplasm (*n* = 3 independent cell culture experiments) (scale bar: 50 μm). (**D**) ATP6V1A decreased lysosomal pH as measured using Dextran, Oregon Green 514 (*n* = 3 independent cell culture experiments). (**E**) Representative double immunofluorescence images of VPS16 (green) and LAMP−1 (red) in H9c2 cells, indicating lysosome−MVBs interaction (scale bar: 10 μm). (**F**) Transmission electron micrographs of H9c2 cells from different treatment groups, with representative images showing MVBs (scale bar: 500 nm). The white arrows pointed to the MVBs. (**G**) Western blot analysis of exosome markers CD63 and TSG101 in H9c2 cell−derived exosomes. Whole−cell lysates were used as controls. (**H**) Exosome concentration measured using AChE activity (*n* = 3 independent cell culture experiments). Data are presented as mean ± SD. * *p* < 0.05 and ** *p* < 0.01 vs. control group.

To further verify the specific role of ATP6V1A in exosome secretion, H9c2 cells were treated with lentivirus overexpressing ATP6V1A (LV-ATP6V1A) or a negative control (LV-NC) following DOX treatment. The successful establishment of ATP6V1A overexpression was confirmed using western blot, and immunofluorescence staining showed increased ATP6V1A expression (Figure 7A,B). ATP6V1A overexpression restored lysosomal acidification capacity, as demonstrated by increased Lysotracker puncta (Figure 7C) and decreased lysosomal pH (Figure 7D). Furthermore, ATP6V1A increased co-localization of the MVB marker VPS16 (green) and lysosome marker LAMP1 (red), as indicated by larger yellow puncta (Figure 7E), and decreased the volume of MVBs in H9c2 cells (Figure 7F). In addition, ATP6V1A reduced the expression of exosomal markers CD63 and TSG101 (Figure 7G) and AChE activity (Figure 7H). These results showed that DOX regulated ATP6V1A expression through SIRT1, leading to inhibited lysosomal acidification and impaired fusion between MVBs and lysosomes, which ultimately promoted exosome release.

### 2.5. DOX-Induced Mitochondrial-Derived Vesicle Formation May Contribute to Exosome Secretion by Cardiomyocytes

**Figure 8:**
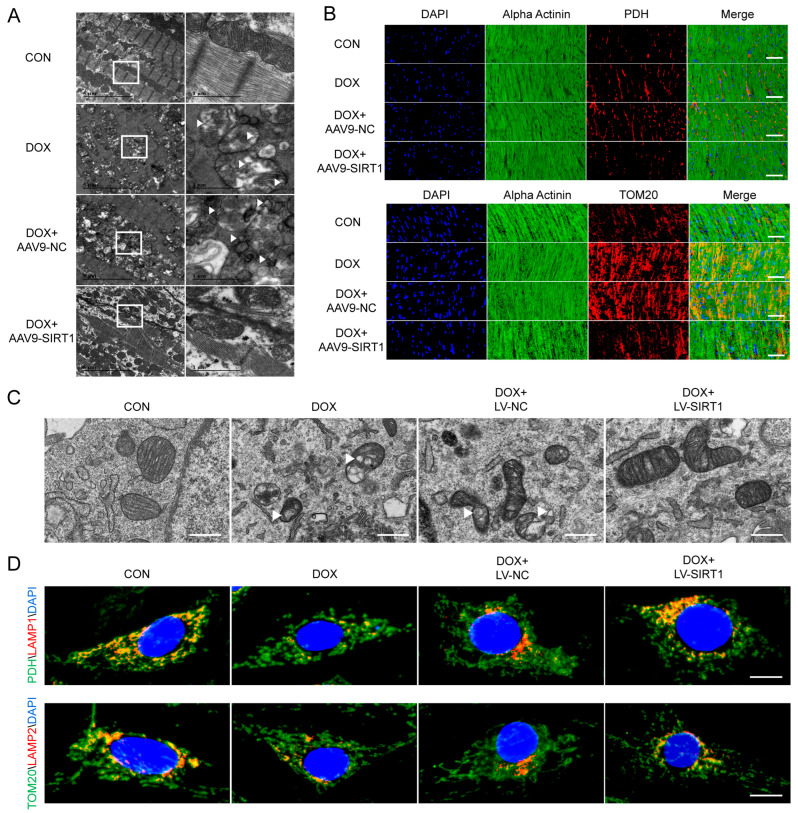
DOX induced mitochondrial−derived vesicle formation. (**A**) Representative transmission electron micrographs of heart tissues. Low magnification images showed general cardiac ultrastructure (scale bar: 5 μm). Higher magnification images revealed mitochondrial morphology and vesicles (scale bar: 1 μm). The white boxes indicated the zoomed−in area shown in the figure on the right. The white triangles pointed to the MDVs. (**B**) Representative immunofluorescence staining showing PDH and TOM20 (red) expression in mouse hearts; both were markers of MDVs (scale bar: 50 μm). Sarcomeric alpha−actinin labeled cardiomyocytes (green). Nuclei stained with DAPI (blue). (**C**) Representative transmission electron micrographs of H9c2 cells depicting mitochondrial morphology and vesicles (scale bar: 500 nm). The white triangles pointed to the MDVs. (**D**) Representative double immunofluorescence images for PDH or TOM20 (green, both were markers of MDVs) with LAMP1\2 (red) in H9c2 cells, indicating interaction between lysosome−TOM20+ or PDH+ MDVs (scale bar:10 μm).

Mitochondrial-derived vesicles (MDVs) are specialized vesicles that bud off from mitochondria and transport specific mitochondria cargo to other cellular compartments, including lysosomes. MDVs can be transported to lysosomes for degradation or to endosomes to form MVBs, from which they are subsequently released extracellularly as exosomes [28]. TEM revealed cardiac mitochondrial injury characterized by swelling and vacuolization with disrupted and lysed cristae in cardiomyocytes treated with DOX (Figure 8A). Notably, MDVs approximately 100 nm in diameter appeared near cardiac mitochondria. Interestingly, SIRT1 overexpression, which restored lysosomal acidification, not only improved cardiac mitochondrial ultrastructural with fewer swollen mitochondria exhibiting cristae loss but also inhibited MDV production. Immunofluorescence staining also showed increased expression of the MDV markers PDH and TOM20 in mouse cardiomyocytes with DOX treatment, while SIRT1 overexpression reduced PDH and TOM20 expression (Figure 8B). Similarly, TEM revealed mitochondrial damage and formation of MDVs with a size of about 100 nm in DOX-treated H9c2 cells (Figure 8C). Co-localization of the MDV markers PDH and TOM20 with the lysosome markers LAMP1\2 decreased with DOX treatment (Figure 8D). However, SIRT1 overexpression restored mitochondrial ultrastructure and inhibited MDV production while increasing PDH/TOM20-LAMP1\2 co-localization. Therefore, DOX may promote lysosomal impairment by downregulating SIRT1 expression, leading to reduced degradation of MDVs and inducing the formation of MDVs in cardiomyocytes.

**Figure 9:**
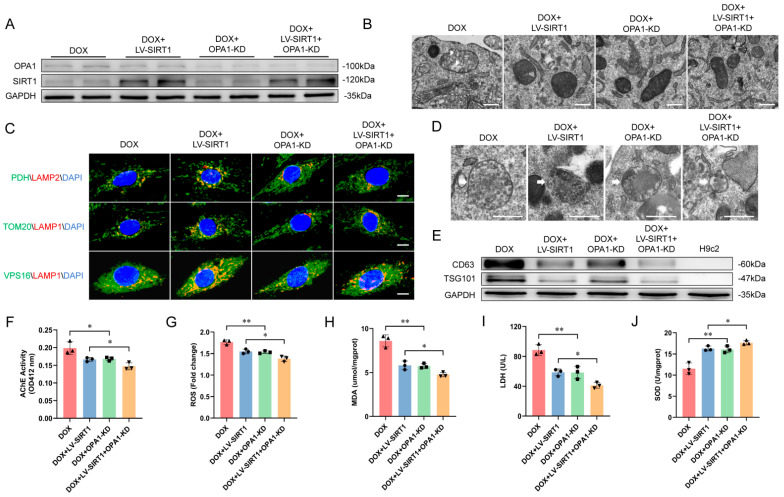
DOX promoted the transport of mitochondria−derived vesicles to MVBs. (**A**) OPA1 and SIRT1 protein expression levels in H9c2 cells measured using western blot. (**B**) Representative transmission electron micrographs of H9c2 cells depicting mitochondrial morphology and vesicles (scale bar: 500 nm). The white triangles pointed to the MDVs. (**C**) Representative double immunofluorescence images of H9c2 cells showing PDH, TOM20, or VPS16 (green) with LAMP1\2 (red), indicating lysosome−TOM20+\PDH+ MDVs or MVBs interaction (scale bar:10 μm). (**D**) Transmission electron micrographs of H9c2 cells from different treatment groups, with representative images showing MVBs (scale bar: 500 nm). The white arrows pointed to the MVBs. (**E**) Western blot analysis of exosomal markers CD63 and TSG101 in H9c2 cell−derived exosomes. Whole−cell lysates were used as controls. (**F**) Exosome concentration measured using AChE activity (*n* = 3 independent cell culture experiments). (**G**–**J**) ROS, MDA, supernatant LDH, and SOD levels in H9c2 cells (*n* = 3 independent cell culture experiments). Data are presented as mean ± SD. * *p* < 0.05 and ** *p* < 0.01 vs. control group.

Optic Atrophy 1 (OPA1) is a key protein that regulates MDV dynamics [29]. Disruption of OPA1 function can lead to alterations in MDV formation and trafficking. To evaluate the effects of MDV production on exosome secretion, we knocked down OPA1 expression in DOX-treated H9c2 cells (Figure 9A). TEM revealed that OPA1 knockdown reduced MDV production compared to DOX treatment alone (Figure 9B). Co-localization of MDV markers PDH\TOM20 and MVB marker VPS16 with lysosome markers LAMP1\2 was unaffected (Figure 9C); however, MVB volume was reduced (Figure 9D). This suggested that inhibition of MDV production may increase their trafficking to MVBs when lysosomal acidification is impaired. In addition, OPA1 knockdown also decreased exosomal markers CD63 and TSG101 expression (Figure 9E) and AChE activity (Figure 9F) while alleviating cell injury (Figure 9G–J). Thus, OPA1 may inhibit exosome release by reducing MDV production and MVB abundance. In DOX-treated H9c2 cells, SIRT1 overexpression exhibited a similar inhibitory effect of OPA1 knockdown. This may be because SIRT1 restored lysosomal acidification to degrade excess DOX-induced MDVs, thereby decreasing MVB abundance and exosome release. In DOX-treated cells with SIRT1 overexpression and OPA1 knockdown cells, these inhibitory effects were further augmented. Therefore, DOX enhanced MDV abundance by impairing lysosomal acidification and reducing MDV degradation, leading to increased MVB abundance and subsequent excess exosome secretion. However, OPA1 silencing and restoration of lysosomal function with SIRT1 overexpression effectively counteracted these deleterious effects of DOX.

## 3. Discussion

Due to the lack of specificity, the off-target effects of DOX not only inhibit the proliferation of tumor cells but also cause cardiomyocyte injury, resulting in severe cardiotoxicity [30]. Conventional views have historically held that DOX induces cell damage through direct cytotoxic mechanisms in cardiomyocytes. However, accumulating evidence now emphasizes potential alternative pathways, such as DOX manipulation of the extracellular microenvironment [19]. As an important component of the microenvironment, exosomes have been recognized as an effective means to understand the physiological and pathological relationships between cardiac components, including cardiomyocytes [31]. Cardiomyocytes release a variety of stress-related proteins [32,33], nucleic acids [34,35], and inflammatory factors [32,36] through exosomes to participate in various cell-related processes. Here, we presented evidence suggesting that DOX induced toxic effects on cardiomyocytes concurrently, leading to excess exosome production. This, in turn, exacerbated cardiomyocyte damage and led to the progression of cardiotoxicity.

In this study, we characterized the cardiotoxic effects of DOX both in vivo and in vitro. Using a mouse model of chronic DOX treatment, we observed impaired cardiac function as evidenced by reduced LVEF and LVFS on echocardiography. Histological examination revealed significant pathological changes to myocardial architecture, including disorganized fibers, interstitial fibrosis, cardiomyocyte hypertrophy, and increased necrosis. Biochemically, DOX increased oxidative stress, as shown by elevated levels of ROS and MDA, increased serum cardiac injury marker LDH content, and decreased antioxidant enzyme SOD activity. Similar toxic effects were reproduced in the H9c2 cardiomyoblast cell line with DOX treatment. This was accompanied by increased ROS, MDA, and supernatant LDH, as well as decreased SOD, paralleling the biochemical changes observed in vivo. These results were consistent with previous studies showing that DOX causes cardiotoxic effects, including impaired cardiac function, structural changes, increased oxidative stress, and cardiomyocyte apoptosis [24,37]. Our findings confirmed that DOX impaired cardiac structure and function by promoting oxidative stress and myocardial damage, as evidenced at both the in vivo and in vitro levels.

According to the guidelines published by the International Society for Extracellular Vesicles (MISEV2018) [38], definitive identification of exosomes requires the detection of at least three exosome-associated proteins, including one transmembrane or lipid-bound protein (such as CD9, CD63 or CD81), one cytosolic protein (such as TSG101, ALIX or HSP70), and exclusion of contamination by other organelles through lack of detection of organelle-specific markers (such as the Golgi apparatus marker GM130 or endoplasmic reticulum marker calnexin). In this study, we observed that DOX treatment led to increased exosome release in H9c2 cells, as evidenced by the elevated expression of the exosomal markers CD9, CD63, TSG101, and AChE activity. We further analyzed the vesicles for the Golgi apparatus marker GM130 by western blot and found it to be absent, fulfilling the third MISEV2018 criterion. This is consistent with previous studies [16,39,40], which have demonstrated that vesicles measuring 100–150 nm in diameter and exhibiting characteristic morphological features and protein markers can be classified as exosomes. While it is possible a very small number of microvesicles may have been present within our exosome isolates, our findings aligned with previous studies indicate that DOX predominantly induced the release of exosomes.

Several reports have indicated that during anti-tumor therapy or induction of cardiac injury, DOX significantly increases serum exosome levels in a time-dependent manner after injection [18,41], which may suggest that exosome release plays a role in the response of cancer cells or cardiomyocytes to DOX, possibly by regulating the accumulation of toxic substances within cells. Exosomes produced by breast cancer cells after DOX or paclitaxel stimulation deliver miR-378a-3p and miR-378d to neighboring cells, inducing chemotherapy-resistant phenotype [42]. In addition to chemoresistance, DOX also resulted in a significant increase in serum circulating exosomes enriched in 4-hydroxynonenal, a lipid peroxidation product associated with DOX-induced cardiotoxicity [41]. In our study, immunofluorescence staining of cardiac tissue from DOX-treated mice showed upregulation of CD63 and TSG101 in cardiomyocytes, suggesting DOX could induce exosome secretion. While exosome release serves to remove pathogenic intracellular substances, this process may overload the extracellular space with toxic molecules, exacerbating harmful effects through uptake, inflammation, and cellular damage [43,44]. Previous studies have linked chemotherapy drugs like DOX to exosome signaling, with chemotherapy-induced exosomes playing an important role in determining cell behavior [18,45,46]. Under DOX treatment conditions, exosomes secreted by cardiomyocytes can act in a paracrine manner on the cells themselves, altering their physiological and pathological states [19]. Similarly, our results showed that incubation of exosomes isolated from the supernatant of DOX-treated H9c2 cells with untreated H9c2 cells promoted the development of an oxidative stress phenotype. The exosome release stimulated by DOX may underlie the mechanism of chemotherapy-induced cardiotoxicity by propagating oxidative damage in the extracellular space.

Correspondingly, blockade of exosome generation has been shown to attenuate inflammation and cardiac dysfunction associated with sepsis [26] and bronchial asthma [47], demonstrating the pathogenetic role exosomes can play under conditions of cellular stress. We thus investigated whether inhibiting DOX-induced exosome production could alleviate cardiomyocyte damage and cardiotoxicity. We found that administration of the exosome inhibitor GW4869 in combination with DOX significantly improved injury both in vitro and in vivo. In H9c2 cells, GW4869 decreased cell injury. Similarly, in mice, GW4869 mitigated DOX-induced cardiac dysfunction, histopathological changes, necrosis, and elevated cardiac oxidative damage. Previous research showed that inhibition of exosome release in vitro and in vivo can reduce the production of proinflammatory cytokines in macrophages and alleviate sepsis-caused cardiac dysfunction [26]. Blocking diabetic exosome secretion from cardiomyocytes also impeded the transfer of miR-320, a well-known anti-angiogenic miRNA, into endothelial cells, counteracting the inhibitory effect on angiogenesis [48]. The enhancement of autophagy flux by rapamycin decreased exosome release from dopaminergic neuronal cells and glial cells under Parkinson’s disease conditions, reducing disease spread and symptoms [49]. Our results showed that DOX stimulated the secretion of proinflammatory exosomes from cardiomyocytes in vivo and in vitro, and inhibition of this exosome secretion effectively alleviated DOX-induced cardiotoxicity.

SIRT1 regulates multiple cellular processes, such as cardiomyocyte survival, metabolic homeostasis, and aging [27,50,51]. Recent studies have pointed out a role for SIRT1 in modulating exosome production and release, suggesting it may affect cellular homeostasis and disease progression via this mechanism [52]. Our study found that DOX decreased SIRT1 expression and induced exosome secretion in H9c2 cells and mouse hearts. SIRT1 overexpression conferred cardioprotective effects against DOX-induced toxicity by reducing exosome secretion in both in vitro and in vivo models. Specifically, SIRT1 overexpression in DOX-treated H9c2 cells significantly reduced exosome release and attenuated cell damage. In mouse hearts, SIRT1 overexpression mitigated the DOX-induced increase in exosome secretion from cardiomyocytes. It considerably lessened cardiac function reduction, tissue injury, necrosis, and oxidative stress. This suggested that SIRT1 overexpression played a protective role against DOX-induced cardiotoxicity, and this protection was mediated, at least partially, by the ability of SIRT to suppress exosome secretion. SIRT1 is a pleiotropic protein that influences a wide range of cellular pathways involved in processes like metabolism, stress responses, and DNA repair [53]. When overexpressed, SIRT1 may activate multiple protective pathways [10,54,55], not necessarily limited to those directly affected by its downregulation. Therefore, even though SIRT1 overexpression reversed DOX-induced damage in our models, this did not definitively prove that the outcomes were solely due to restored exosome secretion levels. Other SIRT1-mediated protective effects, unrelated to its downregulation, may have also contributed to the observed cardioprotective effects. Moreover, the overexpression of the SIRT1 model is unable to isolate the role of SIRT1 from its many complex downstream signaling interactions [11]. Thus, while our data suggest that SIRT1 influences exosome production and chemotherapy response in cardiomyocytes, the protective mechanisms involved likely extend beyond just suppression of exosome release. Recent studies have shown that reduced SIRT1 expression generates a secretome composing excess exosomes with unique cargo and soluble hydrolases that degrade the extracellular matrix, thereby promoting processes that increase breast cancer cell survival and invasion [14]. SIRT1 deletion in senescent stromal cells supports accelerated production of inflammatory exosomes, significantly alters the expression profile of recipient cancer cells, enhances their aggressiveness, and promotes cancer resistance [52]. These studies identify a distinct mechanism by which SIRT1 modulates disease progression by targeting cells and their surrounding microenvironment.

Previous studies have pointed out that loss of SIRT1 impairs proper lysosomal function. Cells lacking SIRT1 expression or activity have difficulty maintaining lysosomal acidification, which makes MVBs less efficient at being degraded in lysosomes and enhances exosome secretion [14,52]. This relates to the fact that SIRT1 loss reduces the expression of ATP6V1A, a subunit of the lysosomal V-ATPase proton pump that mediates lysosomal acidification, leading to impaired acidification [16]. Our study demonstrated that DOX promoted exosome release from cardiomyocytes by decreasing ATP6V1A expression, which limited the fusion of MVBs with lysosomes and led to an increased volume of MVBs. Conversely, SIRT1 and ATP6V1A overexpression were able to reverse these effects of DOX and suppress exosome secretion. Previous work has shown that chemotherapy drug cisplatin treatment significantly reduces the lysosomal compartment in human ovarian carcinoma cells, resulting in abnormal exosome output [56]. Rapamycin enhances lysosomal function and strengthens the interaction between lysosomes and MVBs, which can prevent exosome release from podocytes stimulated by hyperhomocysteinemia and alleviate glomerular inflammation and injury [57]. It is well accepted that the multi-subunit V-ATPase proton pump is essential for maintaining the acidification and function of lysosomes [58]. Reduced levels of the ATP6V1A subunit on lysosomal membranes result in a dysfunctional pump and the inability of lysosomes to maintain the low pH needed for degradation [14]. Notably, DOX treatment has been shown to significantly compromise lysosomal acidification capacity, strongly inhibiting cardiac function over months [22,23]. Here, we showed that DOX reduced the expression of ATP6V1A through SIRT1, impairing the acidification of lysosomes. Through this process, the degradation of MVBs in lysosomes was inhibited, and exosome secretion in cardiomyocytes was promoted, aggravating cardiotoxicity.

Our study observed, both in vivo and in vitro, signs of mitochondrial damage in DOX-treated cardiomyocytes, as characterized by obvious mitochondrial swelling, vacuolization with disintegration, and cristae degradation. Under stress conditions, mitochondria produce MDVs in the range of 70–150 nm labeled with either positive markers PDH (mitochondrial matrix) or TOM20 (mitochondrial outer membrane) prior to mitochondrial depolarization [59,60,61]. This suggests that MDV formation represents an essential housekeeping mechanism and the first line of defense against mitochondrial stress. Consistent with previous studies, we detected MDVs averaging 100 nm surrounding cardiomyocyte mitochondria in DOX-treated mouse hearts. This was accompanied by increased expression of PDH and TOM20. Similarly, in vitro, DOX treatment caused mitochondria to produce MDVs averaging 100 nm in size. Previous studies have shown that the fate of MDVs is related to the lysosomal integrity and the transport of MDVs to lysosomes for degradation [62,63]. Our study found that SIRT1 substantially reduced MDV production and PDH/TOM20 expression in cardiomyocytes in vivo and in vitro while increasing their co-localization with lysosomes in H9c2 cells. Reduced or absent SIRT1 can disrupt the normal functioning of lysosomes and act as a compensatory mechanism to dispose of the accumulated, undigested cellular components, such as MDVs, which are normally degraded within the lysosomes. However, DOX compromised lysosomal acidification by inhibiting SIRT1 expression, thereby promoting the accumulation of MDVs in H9c2 cells, which may contribute to exosome release.

In addition to degradation in lysosomes, some MDVs specifically fuse with endosomes to form MVBs, which are then released as exosomes into the extracellular environment [28]. Our study found that OPA1 knockdown and SIRT1 overexpression both reduced MDV production in DOX-treated cells. OPA1 inhibition may decrease MDV trafficking to MVBs when lysosomal acidification is impaired by DOX to stimulate exosome release. SIRT1 overexpression mimicked the inhibitory effects of OPA1 knockdown by restoring lysosomal acidification to degrade excess DOX-induced MDVs, further reducing MVBs and restricting exosome secretion. Combined SIRT1 overexpression and OPA1 knockdown further augmented this inhibitory effect. Moreover, increased exosomes carrying low amounts of mitochondrial components are found in the serum of patients with Parkinson’s or physical frailty and sarcopenia compared with healthy controls [64,65], supporting that MDVs are a potential biological source of exosomes. Under stress, mitochondrial dysfunction may suppress lysosomal function in neurons and glial cells, leading to decreased autophagic flux and increased exosome release [49]. Therefore, interorganellar crosstalk between mitochondria, lysosomes, and MVBs appears critical in DOX-induced cardiotoxicity pathogenesis. Both enhanced MDV biogenesis and dysregulated lysosomal function likely contributed to robust exosome release from stress.

Clinical and preclinical data consistently demonstrate that gender is an important risk factor for DOX-induced cardiotoxicity [66]. Numerous studies have demonstrated sexual dimorphism, with male rodent models generally exhibiting greater susceptibility to both acute and chronic DOX-induced cardiotoxicity compared to females [67,68,69,70,71]. Sex hormones have been shown to modulate the severity of DOX cardiotoxicity [66,72,73], with male hormones potentially increasing vulnerability and female hormones providing some degree of protection. These collective factors contribute to marked sex-related differences in DOX-induced cardiotoxicity. Therefore, it is important to include both genders in future research on DOX-induced cardiotoxicity to facilitate a more comprehensive analysis of gender-related toxic effects and underlying mechanisms.

In conclusion, this study shows that DOX promotes excess exosome secretion from cardiomyocytes by downregulating SIRT1. SIRT1 suppression inhibits the expression of the V-ATPase subunit ATP6V1A, thereby impairing lysosomal acidification and reducing the fusion of MVBs with lysosomes. Meanwhile, DOX triggers mitochondrial stress, leading to increased MDV production, potentially transporting cargo to MVBs and increasing their abundance. These pathological factors eventually result in exosome release. The secreted exosomes then propagated additional damage to neighboring cardiomyocytes, exacerbating DOX-induced cardiotoxicity. This study uncovered a novel pathophysiological pathway by which DOX-induced cardiomyocyte injury occurs through exosome mediation and elucidates that targeting the regulatory pathway of exosome release from cardiomyocytes may be a potential therapeutic strategy for DOX-induced cardiotoxicity.

## 4. Materials and Methods

### 4.1. Animals and Experimental Protocols

Male 7-week-old C57BL/6J mice weighing 18–20 g were purchased from the Shanghai Model Organisms Center (Shanghai, China) and housed under standard conditions of controlled humidity and a 12 h light/dark cycle at 22 °C. After a 1-week acclimation period, mice were randomly assigned to four groups (*n* = 6): (I) control group, (II) GW4869 group (i.p., 2.5 μg/g; Cat#HY-19363, MedChemExpress, Monmouth Junction, NJ, USA), (III) DOX group (i.p., 5 mg/kg/week; Cat#HY-15142, MedChemExpress, USA), and (IV) GW4869 + DOX group. DOX was injected intraperitoneally once a week for 4 consecutive weeks, with GW4869 administered 1 h in advance [26], while the control group was given the same amount of saline. To overexpress SIRT1 specifically in the myocardium, one week following the final DOX injection, mice received a single intravenous injection of adeno-associated virus 9 (AAV9) carrying SIRT1 (AAV9-SIRT1) or a negative control (AAV9-NC) (GenePharma, Shanghai, China) at a concentration of 1 × 10^12^ viral genome per mouse via the tail vein. 2 weeks after AAV9 injection, echocardiography was performed, and blood/heart tissue samples were obtained from mice anesthetized with deep isoflurane (5%) and stored at −80 °C for subsequent analyses.

### 4.2. Echocardiography

Transthoracic echocardiography was performed using a 30 MHz high-frequency scan probe (Vevo 2100, VisualSonics, Toronto, ON, Canada). Mice were anesthetized with 1–3% isoflurane and maintained at 2 L/min 100% oxygen during the procedure. Blind echocardiographic measurements were taken as previously described [74]. Left ventricular ejection fraction (EF) and fractional shortening (FS) were measured and calculated using Vevo Analysis software (Vevo 2100. Imaging System software version 1.0.0. Printed in Canada).

### 4.3. Histological Analysis

Heart tissues were fixed in 4% paraformaldehyde and embedded in paraffin. Sectioned were then cut from the paraffin-embedded hearts and stained with hematoxylin-eosin (H&E) staining (Cat#G1005, Servicebio Biotech, Wuhan, China), Masson’s trichrome staining (Cat#G1006, Servicebio Biotech, China), wheat germ agglutinin (WGA) staining (Cat#G1730, Servicebio Biotech, China), and terminal deoxynucleotidyl transferase dUTP nick-end labeling (TUNEL) staining (Cat#G1504, Servicebio Biotech, China) assays according to manufacturer protocols. Images were observed and captured with a Nikon Eclipse Ti fluorescence microscope (Nikon, Tokyo, Japan) and processed using Image J software (Version 1.54k).

### 4.4. Immunohistochemistry and Immunofluorescence Staining

For immunohistochemistry staining, heart sections were deparaffinized by incubation with xylene and rehydrated in an ethanol gradient. Sections were washed with phosphate-buffered saline (PBS), and endogenous peroxidase activity was quenched by incubating sections with 3% H_2_O_2_ in methanol for 10 min. Sections were blocked for 1 h at 37 °C with 5% goat serum (Cat#C0265, Beyotime Biotechnology, Haimen, China). Primary antibody incubation (Table S1) was conducted overnight at 4 °C. Sections were incubated for 1 h at room temperature with horseradish peroxidase (HRP)-conjugated anti-rabbit secondary antibody (Cat#GB23303, 1:500, Servicebio Biotech, Wuhan, China). Control slides were stained with isotype IgG to verify antibody specificity. Immunoreactivity was visualized using 3,3`-diaminobenzidine (DAB) chromogen solution for 5 min and rinsed with PBS.

For immunofluorescence analysis, heart sections or H9c2 cells were fixed with 4% paraformaldehyde for 20 min at room temperature. After rinsing with PBS, sections/cells were blocked for 1 h at 37 °C with PBS containing 10% goat serum (Cat#C0265, Beyotime Biotechnology, China) and 0.3% Triton-100 (Cat#9036-19-5, Sigma-Aldrich, St. Louis, MO, USA). Sections/cells were incubated overnight at 4 °C with primary antibodies (Table S1), followed by incubation for 1 h at room temperature with Alexa Fluro-conjugated secondary antibodies (Table S1). Nuclei were stained with 4′,6-diamidino-2-phenylindole (DAPI) for 10 min. Image acquisition was performed using a Nikon Eclipse Ti fluorescence microscope (Nikon, Japan).

### 4.5. Cell Treatment and Transfection

H9c2 rat cardiomyocytes were purchased from the Type Culture Collection of the Chinese Academy of Sciences (Shanghai, China) and cultured in Dulbecco’s modified Eagle’s medium (DMEM; Cat#11965092, Hyclone, Logan, UT, USA) supplemented with 10% fetal bovine serum (FBS; Cat#10099141, Gibco, Norristown, PA, USA) and 1% penicillin–streptomycin solution (Cat#15070063, Gibco, USA) at 37 °C in a humidified incubator with 5% CO_2_. Cells were seeded in 6-well plates and replaced serum-free with DMEM when reaching 80% confluence. DOX treatment was performed at concentrations of 0–10 μM for 24 h or 1 μM for 0–48 h. To suppress exosome secretion, H9c2 cells were pretreated with 20 μM GW4869 for 2 h [26] prior to incubation with 1 μM DOX for 24 h.

Lentivirus overexpressing SIRT1\ATP6V1A (LV-SIRT1\ATP6V1A) or a negative control (LV-NC) (GenePharma, Shanghai, China) were transduced at 1 × 10^8^ transfection units per milliliter. Transfected cell clones designated LV-SIRT1\ATP6V1A or LV-NC were seeded at a density of 1 × 10^5^ cells/mL. Following overnight incubation, lentivirus and polybrene were added when cells reached 70% confluence. Medium was replaced 24 h later, and transfection efficiency was assessed after 72 h using an inverted phase/fluorescence microscope (Olympus, Tokyo, Japan). H9c2 cells were transfected with OPA1 siRNA (sense: CCAGCAAGGUUAGCUGCAATT; antisense: UUGCAGCUAACCUUGCUGGTT) (GenePharma, China) or negative control using Lipofectamine RNAiMAX reagent (Invitrogen, Waltham, MA, USA) according to the manufacturer’s protocol. siRNA effects were confirmed using a western blot.

### 4.6. Exosome Purification and Characterization

For exosomes isolation, H9c2 cells were divided into four groups and treated in exosome-free FBS medium for 24 h: (I) control, (II) 20 uM GW4869, (III) 1 uM DOX, and (IV) GW4869 + DOX. Exosomes were also isolated from DOX-treated H9c2 cells transfected with lentivirus overexpressing SIRT1\ATP6V1A or transfected with siRNA targeting OPA1. The conditioned medium used for exosome extraction was normalized to cell numbers. Exosomes were purified by ultracentrifugation as described [75]. Briefly, cell supernatant was filtered with a 0.22 μm syringe filter to remove cells and debris, then subjected to sequential centrifugation at 300× *g* for 10 min, 2000× *g* for 10 min, 10,000× *g* for 30 min, and 100,000× *g* for 70 min. The pellet was washed in PBS and again centrifuged at 100,000× *g* for 70 min (Optima XPN-100, Beckman, Indianapolis, IN, USA). Exosome pellets were resuspended in PBS and stored at −80 °C. Transmission electron microscopy and nanoparticle tracking analysis were used to validate intact exosome morphology and measure size distribution. Protein concentration was determined using a bicinchoninic acid protein assay kit (Cat#P0010S, Beyotime Biotechnology, China). Isolated exosomes were fluorescently labeled with 3,3′-dioctadecyloxacarbocyanine perchlorate (DiO) kit according to manufacturer’s protocols (Cat# C1038, Beyotime Biotechnology, China).

### 4.7. Transmission Electron Microscopy

For transmission electron microscopy morphological analysis, freshly excised heart tissues or cell pellets were fixed in 2.5% glutaraldehyde in 0.1 M sodium cacodylate buffer (pH 7.4) at 4 °C for 2 h. Samples were washed with 0.1 M sodium cacodylate buffer and post-fixed with 1% osmium tetroxide for 2 h at 4 °C. Dehydrated was performed using a graded ethanol series at 4 °C, followed by infiltration and embedding with epoxy resin at room temperature over 4 h. Ultra-thin sections (70 nm) were cut and transferred to copper grids before imaging using transmission electron microscope (Hitachi, Tokyo, Japan).

### 4.8. Cell Viability

H9c2 cell viability was assessed using the enhanced Cell Counting Kit-8 (CCK-8; Cat#C0042, Beyotime Biotechnology, China). Cells were seeded at 5000 cells/well in 96-well plates and treated as described above. Following treatment, CCK-8 working solution was added to each well and incubated for 2 h at 37 °C. Absorbance was measured at 450 nm using a microplate reader to determine cell viability in each treatment group.

### 4.9. Biochemical Assays

Reactive oxygen species (ROS) were detected following the manufacturer’s instructions (Nanjing Jiancheng Bioengineering Institute, Nanjing, China). Briefly, when H9c2 cells reached 70–80% confluency, cells were treated with different drugs and subsequently incubated with the ROS working reagents at 37 °C for 45 min in the dark. For heart tissue ROS detection, cardiomyocytes were first isolated from fresh heart tissues via collagenase II digestion, following which ROS detection was performed using the working reagents. ROS levels were then measured in a microplate reader at 525 nm. Commercial assay kits (Beyotime Biotechnology, China) were used to measure malondialdehyde (MDA), superoxide dismutase (SOD), and lactate dehydrogenase (LDH) according to the manufacturer’s protocols. For MDA and SOD, measurements were made in heart tissue homogenates and H9c2 cell lysates. LDH was detected in serum and H9c2 cell culture supernatants.

### 4.10. Lysosomal pH Measurement

Lysosomal pH was quantitatively assessed, as described previously, with modifications [76]. Briefly, H9c2 cells were plated in 35 mm dishes (NEST Biotechnology, Wuxi, China) and loaded with 100 μg/mL of Dextran-conjugated Oregon Green 514 for 2 days prior to DOX treatment. Following treatment, cells were washed with PBS and incubated with physiological buffer (136 mM of NaCl, 2.5 mM of KCl, 2 mM of CaCl_2_, 1.3 mM of MgCl_2_, 5 mM of glucose, 10 mM of HEPES at pH 7.4) for 1 h at 37 °C. Confocal imaging was performed using an Andor Spinning Disk confocal microscope with an Apo TIRF 60x/1.49 NA Oil lens, Nikon Ti stand, and Nikon Perfect Focus system. Dextran–Oregon Green 514 was excited at 445 nm and 488 nm, with emitted light filtered at 525 ± 40 nm. Calibration curves were generated by sequentially bathing nigericin-treated cells in isotonic K^+^ solutions (145 mM of KCl, 10 mM of glucose, 1 mM of MgCl_2_, and 20 mM of HEPES) of pH 4.0–6.5. The 488/445 emission ratios for each cell were analyzed using Image J software) and plotted versus pH. Sample ratios were interpolated using a calibration curve from three independent experiments of 100–120 cells each.

### 4.11. Western Blot Analysis

Total protein was extracted from cultured cells or frozen cardiac tissues using radioimmunoprecipitation (RIPA) lysis buffer supplemented with protease and phosphatase inhibitor cocktail (Cat#P1050, Beyotime Biotechnology, Haimen, China). Lysates were clarified by centrifugation at 14,000× *g* for 15 min at 4 °C. Protein concentration was determined using a bicinchoninic acid assay (Cat#P0010S, Beyotime Biotechnology, China). Equal amounts (20 μg) of protein were separated by sodium dodecyl sulfate polyacrylamide gel electrophoresis (SDS-PAGE) and transferred onto polyvinylidene difluoride membranes (Millipore, Burlington, MA, USA). Membranes were blocked for 2 h with 5% DifcoTM Skim Milk (Cat#232100, BD Biosciences, San Jose, CA, USA) and probed overnight at 4 °C with primary antibodies (Table S1). Horseradish peroxidase-conjugated goat anti-rabbit IgG or goat anti-mouse IgG (1:3000 dilution; Cat#M21002/M21001, Abmart, Shanghai, China) were incubated for 1 h. Protein bands were visualized and quantified using an Odyssey Infrared Imaging System.

### 4.12. Assessment of Acetylcholine Esterase Activity

The quantity of exosomes in cell culture supernatants was indirectly estimated by measuring acetylcholine esterase (AChE) activity, as AChE is enriched in exosomal membranes [25,26]. AChE activity was determined using a commercial kit (Cat#MAK119, Sigma-Aldrich, USA) according to the manufacturer’s protocol. Culture supernatants were incubated with the working reagent in 96-well plates. Following incubation, the absorbance of the colorimetric product was read at 412 nm using a microplate reader. AChE activity was calculated in units per liter based on the standard curve, as per kit instructions. This assay provided a quantitative measure of exosomal content in supernatant samples.

### 4.13. Statistical Analysis

Data are presented as mean ± SD. For in vivo experiments, at least six mice were used per group. For in vitro experiments, assays were independently replicated for a minimum of three times. Comparisons between groups were made using an unpaired two-tailed Student’s *t*-test. For multiple group comparisons, one-way ANOVA with Tukey’s post-hoc test was used. Statistical analyses were performed using GraphPad Prism 8.0. Differences with *p* < 0.05 are considered statistically significant.

## Data Availability

The data and materials used to support the findings of this study are available from the corresponding author upon request.

## Supplementary Materials

The following supporting information can be downloaded at: https://www.mdpi.com/article/10.3390/ijms252212376/s1.
