# Supplementary material for: Doxorubicin-Induced Cardiotoxicity Through SIRT1 Loss Potentiates Overproduction of Exosomes in Cardiomyocytes
Source: Int J Mol Sci. 2024 Nov 18;25(22):12376. doi: 10.3390/ijms252212376 (PMC11594621; doi:10.3390/ijms252212376)
Supplement: Supplementary file 1 [file ijms-25-12376-s001.zip › ijms-3261071-supplementary.pdf]

Table S1: Antibodies Used for Western Blot Analysis and Immunohistochemistry and Immunofluorescence Staining.

Antibodies	Cat. No. and Company	Dilution Ratio
CD63	sc-5275, Santa Cruz	1:1000 for WB, 1:200 for IF
TSG101	ab125011, Abcam	1:1000 for WB, 1:100 for IF
GM130	A5344, ABclonal	1:1000 for WB
GAPDH	AC002, ABclonal	1:5000 for WB
SIRT1	8469T, Cell Signaling Technology	1:1000 for WB, 1:100 for IF, 1:200 for IH
ATP6V1A	ab199326, Abcam	1:2000 for WB, 1:250 for IF, 1:1000 for IH
VPS16	17776-1-AP, Proteintech	1:100 for IF
LAMP1	GTX13523, GeneTex	5-10 μg/mL for IF
LAMP2	GB11330-100, Servicebio	1:500 for IF
Alpha Actinin	ab137346, Abcam	1:500 for IF
PDH	ab110334, Abcam	1:1000 for WB, 1:1000 for IF
TOM20	ab186734, Abcam	1:1000 for WB, 1:250 for IF
OPA1	ab119685, Abcam	1:1000 for WB
Goat Anti-Rabbit IgG H&L	ab150077, Abcam	1:200 for IF
Goat Anti-Mouse IgG H&L	ab150114, Abcam	1:200 for IF
